# Developmental trajectories of body mass index and emotional-behavioral functioning of underweight children: A longitudinal study

**DOI:** 10.1038/srep20211

**Published:** 2016-01-25

**Authors:** Silvia Cimino, Luca Cerniglia, Carlos A. Almenara, Stanislav Jezek, Michela Erriu, Renata Tambelli

**Affiliations:** 1 Sapienza, University of Rome. Psychology and Medicine Faculty, Department of Dynamic and Clinical Psychology. Via dei Marsi, 78–00186, Rome, Italy; 2 International Telematic University Uninettuno, Psychology Faculty, Department of Psychology, Corso Vittorio Emanuele II, 39–00100, Rome, Italy; 3 Institute for Research on Children, Youth and Family, Faculty of Social Studies, Masaryk University, Jostova 10, Brno, Czech Republic; 4 Italian Center for Relational Psychotherapy, Viale Regina Margherita, 269–00198, Rome

## Abstract

Although several studies have addressed developmental trajectories from childhood to adolescence of internalizing/externalizing problems, limited attention has been given to underweight children. Two groups were recruited for this study from a community sample: underweight (Ug, N = 80, 50% female) and normal weight (NWg, N = 80, 50% female) to examine the developmental trajectories of body mass index and emotional-behavioral functioning of underweight children from the age two years, and their risk of eating disorder at early adolescence. The study was organized over four waves, each of three years. Pediatricians measured BMI, parents completed the Child Behavior Checklist (CBCL) and the Eating Disorders Inventory-Referral Form (EDI-3-RF). Our results showed that children in the two groups recorded different BMI trajectories over time. In NWg, male and female subjects started from a higher BMI at T1 than their peers. In Ug, internalizing and externalizing problems in males and females remained higher than their peers at all points of assessment. Males and females in Ug scored higher than those in NWg on EDI-3-RF total score. Our results indicate a need for effective physical and psychological assessment of underweight children in community samples to prevent psychological difficulties and eating disorders in adolescence.

## Introduction

Low body weight and malnutrition during childhood have been acknowledged internationally as a significant public health problem, associated with illiteracy, poverty, and maternal mortality among other factors in underdeveloped and developing countries^1^. By contrast, in developed countries where food is largely available, low body weight among children is sometimes associated with non-organic etiopathogenesis. In Europe and Western countries, prevalence of low body weight and low height in children from two years of age to adolescence varies from 1.5% to 14%^2^,^3^. In the present paper, we will refer to *underweight* as defined by the International Obesity Task Force (IOTF), which proposed an international Body Mass Index (BMI, kg/m^2^) reference standard to define underweight, overweight, and obesity^4^,^5^, according to subjects’ height and age. Further, we consider underweight subjects whose mothers experienced no peri- or post-partum difficulties or health problems, who were not born preterm, and were of average socioeconomic status^6^,^7^. The gravest complications of underweight arise when insufficient food intake results in failure to thrive or in stunted growth. It has also been suggested that brain development depends on adequate nutritional intake, especially for the myelinization of axons and the construction of neuronal networks critical for motor control and the regulation of emotions^8^,^9^. It has also been proposed that early childhood malnutrition may lead to health problems such as arterial hypertension and cardiac disease in later life^10^,^11^. A number of studies have also demonstrated that, paradoxically, obesity can emerge later in life, although the mechanism underpinning this phenomenon has not yet been clarified^12^,^13^,^14^. Moreover, some authors have found clinical links and continuity between underweight during childhood and eating disorders in adolescence, which can occur at different developmental stages and particularly in early childhood and/or during puberty^15^,^16^. In particular, many studies have shown maladaptive relational patterns between mothers and their underweight children in their first two years of life, with poor dyadic emotional attunement, lack of empathy, and insecure patterns of attachment^17^,^18^,^19^,^20^. Conversely, there is a dearth of literature and empirical studies (from early development to early and mid-adolescence) on trajectories of behavioral-emotional functioning of underweight children^21^.

### Trajectories of internalizing/externalizing problems in underweight children

Emotional and behavioral problems in children have been widely examined by the evaluation of internalizing and externalizing functioning. Internalizing problems of early childhood typically take the form of withdrawal, anxiety, fearfulness, and depression; externalizing problems commonly take the form of explicit disruptive or oppositional behaviors such as aggression, defiance, and hyperactivity. While interest in the developmental trajectories of these problems has prompted a growing research literature over the last decade, relatively limited attention has been given to the individual-level developmental trajectories of children with low BMI. Conversely, a number of studies focusing on children with high BMI have suggested that these children show more maladaptive internalizing and externalizing scores. In particular, trajectories of emotional functioning in these subjects show externalizing symptoms in males and internalizing problems in females to a greater extent than among subjects with normal BMI^22^.

The few studies that have focused on underweight children have used clinical samples, mostly involving mothers with a psychiatric diagnosis (typically, eating disorders) or focusing on emotional dysregulation as a consequence of preterm birth^23^,^24^,^25^. These studies found that behavioral and emotional difficulties in preschool are often a prelude to psychopathology in middle childhood and beyond^26^,^27^,^28^,^29^. In clinical samples with underweight children, early emotional and behavioral problems have been found to lead to child, adolescent, and adult antisocial and depressive psychopathologies. It is therefore of great importance to identify at an early stage children from the community who may be at risk of high and ongoing internalizing and externalizing problems^30^,^31^.

### The present study

We examined both internalizing and externalizing problems within a developmental psychopathology-based conceptual framework—that is, concerned with individual differences in the origins, course, and outcomes of normative and psychopathological developmental processes^31^,^33^. In this paper, we adopt a primary prevention framework to observe and describe low BMI and emotional-behavioral functioning trajectories from 2 to 11 years of age.

The present research has three specific objectives: to describe the stability and change of the BMI from age 2 to 11 years in two groups of children (underweight and normal weight) of both sexes; to describe the development over time of emotional-behavioural functioning in both groups; and to examine the risk of eating disorder at age 11 in both groups.

## Methods

### Sample and procedure

In 2004, our research group commenced a screening program in collaboration with paediatricians working in public and private schools in Central Italy. The aim was to detect underweight children and to examine their trajectories over time for BMI and emotional-behavioral functioning. The study protocols were approved by the Ethical Committee of the Psychology Faculty of Sapienza—University of Rome, in accordance with the guidelines approved in Helsinky Declaration. Additionally, participating schools obtained ethical clearance through their respective institutional review bodies. The study was organized over four waves (T1 to T4), each of three years. Our collaborating group of paediatricians and psychologists executed the physical and psychological assessments (see Tools section below). At T1, in 2004, over a period of 6 months, we recruited 80 families. The sample of children was balanced by sex and randomly selected using computer software from among families with two-year-old underweight children below the third percentile for weight (Underweight group/Ug), evaluated by paediatricians in schools on the basis of World Health Organization (WHO) growth curves^5^ in the absence of any referred medical and/or psychiatric diagnosis. Ug was paired to a sample of N = 80 families (randomly selected) in which two-year-old children presented with adequate growth and without further medical and/or psychiatric impairment (Normal Weight group/NWg). Informed consent was obtained from all subjects for the aims of the study, and at each wave, both parents completed a scale measuring their child’s emotional-behavioral functioning (see Tools section below). This procedure was repeated for each wave, and the sample was not affected by attrition. At T4, with parents’ written consent, early adolescents were asked to fill out a questionnaire designed to assess risk of eating disorder.

## Tools

### Body Mass Index (BMI)

The BMI is a composite measure of children’ weight, height, and age. International cut-off points were used to identify underweight children^5^.

### Children’s internalizing/externalizing problems

For this purpose, we used the two Italian versions of the Child Behavior Checklist (CBCL)^34^: one designed for children of 1½–5 years and a second for ages 6–18 years^35^. Both versions include items that assess internalizing/externalizing problems, although the formulation of some items differs across versions to ensure coherence with developmental changes within these domains. As this study uses growth curve modeling, we needed to ensure measurement equivalence across time points^36^. In line with the study of Gilliom and Shaw^30^, we adopted internalizing/externalizing items that are included on both versions of the CBCL. Nine items were chosen to represent externalizing problems: “Can’t sit still, restless, or hyperactive;” “Cruel to animals;” “Destroys his own things;” “Destroys things belonging to his family or others;” “Disobedient;” “Doesn’t seem to feel guilty after misbehaving;” “Get in many fights;” “Physically attacks people;” and “Temper tantrums or hot temper.” Six items were chosen to represent internalizing problems: “Too fearful or anxious;” “Self-conscious or easily embarrassed;” “Shy or timid;” “Unhappy, sad, or depressed;” “Withdrawn, doesn’t get involved with others;” and “Worries.” The CBCL employs a 3-point Likert scale: 0 = *not true*, 1 = *somewhat or sometimes true*, and 2 = *very true or often true*. Internal consistency was high for the externalizing scale (Cronbach’s α = 0.78 to 0.82 across time points) and moderate for the internalizing scale (Cronbach’s α = 0.72 to 0.78).

### Early adolescents’ risk of eating disorder

The EDI-3 Eating Disorders Inventory—Referral Form (EDI-3-RF)^37^ (Italian version)^38^ is a 25-item brief self-report measure, derived from the EDI-3 (which contains 91 items) and designed to measure eating disorder risk. The EDI-3-RF can be administered in non-clinical or clinical settings and, as indicated by the scale’s author and by other studies (e.g.^39^), the measure can be administered to subjects as young as eleven years old. The EDI-3-RF uses a 6-point Likert scale (1 = *always*, 6 = *never)* forming the Drive for Thinness (e.g., “I think about dieting”), Bulimia (e.g., “I eat moderately in front of others and stuff myself when they’re gone”), and Body Dissatisfaction subscales (e.g., “I think that my stomach is too big”).

## Analyses

Preliminary screening of the data showed few missing data for each instrument (3%). Missing data were corrected using multiple imputation. We used the MIXED and GENLINMIXED procedures in IBM SPSS 22 to estimate multilevel growth curve models and CBCL trajectories for the internalizing and externalizing subscales. Although we considered using a single multivariate growth model, the limited sample size meant it was not possible to achieve stable estimates of the random effect parameters, even in the univariate models. Ordinary univariate factorial ANOVA was also used.

## Results

### Demographic characteristics of the sample

In Ug and NWg, 94% and 92% respectively of children were firstborn, 97% and 93% of households were intact, and all the children were of homogeneous nationality and were their parents’ biological children. Most families were of middle socioeconomic status (93%)^6^. All female subjects in both Ug and NWg reached menarche at T4. Table 1 presents basic descriptive statistics for individual variables. While BMI was approximately normally distributed in our sample, the distribution of both CBCL scales was markedly positively skewed for both Ug and NWg. The EDI-3 scales showed a small positive skew in NWg but stayed symmetrical in Ug. It should be noted that the distributions of EDI-3-RF scales in Ug and NWg did not overlap and were completely discrete.

### Developmental trajectories of BMI

**Figure 1:**
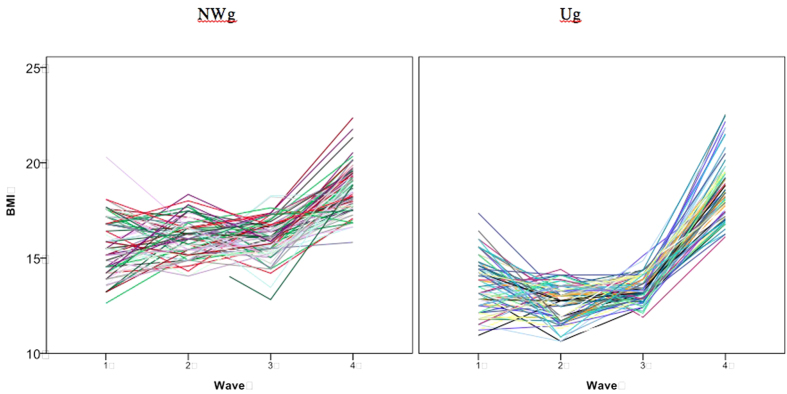
Individual BMI growth curves in NWg and Ug.

A growth curve modelling approach (as described by Singer and Willet^40^) was used to capture BMI developmental trajectories. In these models, age is treated as a continuous variable. The small sample size allows all the individual empirical growth curves to be displayed (Fig. 1). In NWg, the individual trajectories started higher than in Ug and remained higher until the last wave, where Ug caught up. It can also be observed that BMI variance is highest in waves 1 and 4.

In contrast to the between-groups differences, the differences between individuals within groups appear much less systematic. This is also evidenced by the very low correlation between BMIs across waves (see Table 2) in the two contrast groups, and it appears that the most suitable conceptualization in terms of the growth curve model would be that individual growth curves are a random deviation from a common trend specific to that group.

**Figure 2:**
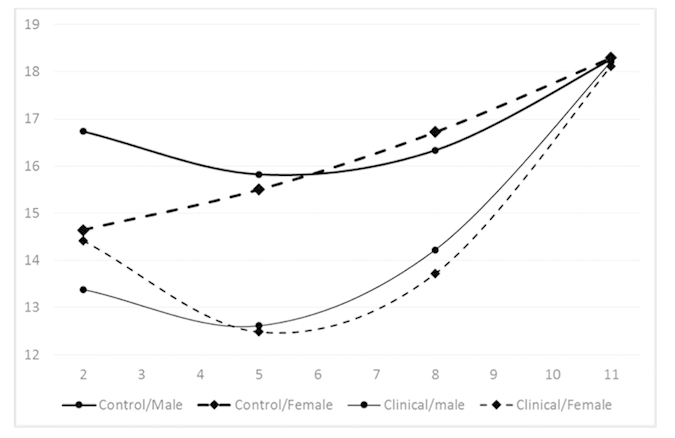
Modeled mean BMI growth curves by group and sex. Note: Curves represent mean developmental trajectories.

Based on the observed growth trajectories, a growth curve model was specified by linear and quadratic trend, conditioned on group membership in Model 1 and on group membership and sex in Model 2. Because of the small sample and low systematic differences between individuals in both groups, the stochastic part of the model was fully fixed—that is, the variance of growth parameters was zero, and the model had no random effects. The results of the growth curve modelling of BMI are reported in Table 3. Both models capture most of the variance in BMI across waves and individuals (Model 1 captures 75%, and Model 2 captures 78%). The inclusion of sex significantly improved the model, both in terms of information criteria and of interpretability. To facilitate interpretation, mean growth curves are plotted in Fig. 2.

### The development of internalizing/externalizing problems

**Figure 3:**
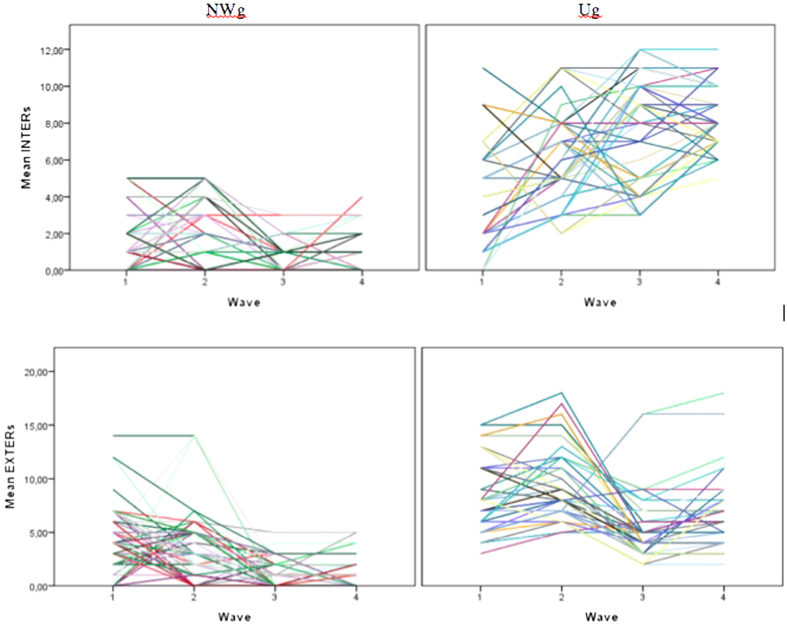
Individual internalizing/externalizing growth curves for NWg and Ug.

To describe the development of internalizing/externalizing problems as measured by CBCL over the span of the study, we used the same multilevel approach as for BMI. Individual empirical growth curves are presented in Fig. 3. NWg trajectories differ visibly from Ug trajectories for both internalizing and externalizing problems; the level of both tends to be lower in NWg. In Ug, we can see a heterogeneous set of trajectories; the internalization trajectories of Ug are particularly variable. In NWg, the trajectories for both internalization and externalization tend to decrease, unlike the Ug group.

Wave-to-wave correlations of reported internalizing/externalizing problems showed a similar pattern. Correlations between the last two waves were positive and high; correlations between the first two waves were small (other than for externalization) but still positive. In Ug, there were small negative correlations between waves 1 and 3, and 4 (see Table 4).

Because both internalization and externalization were positively skewed, we specified the growth curve model as a generalized multilevel model, assuming negative binomial distribution with log link function. The models include a linear trend conditioned on group membership in Model 1 and on group membership and sex in Model 2. Again, the stochastic part was fully fixed in all models. A summary of the models is presented in Table 5.

**Figure 4:**
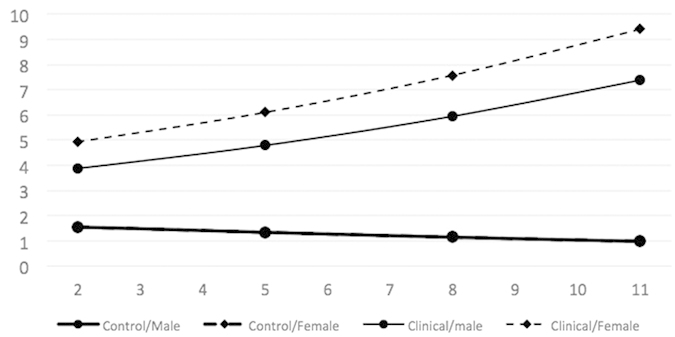
Modelled mean internalization growth curves by group and sex. Note: Curves represent mean developmental trajectories. Male and female trajectories in NWg overlay each other and appear as one trajectory.

Both models of internalization captured a large proportion of variance in internalization scores across waves and individuals; Model 1 captured 72%, and Model 2 (surprisingly) captured slightly less (71%). While the inclusion of sex significantly improved the model in terms of information criteria, the model was unable to differentiate between various forms of sex terms—main effect, interaction with age or group, and interaction with age and group—suggesting that the data are insufficient to determine how best to describe the effect of sex. At the same time, information criteria suggest that the inclusion of sex in any way improves the model. To facilitate interpretation, mean growth curves are presented in Fig. 4.

**Figure 5:**
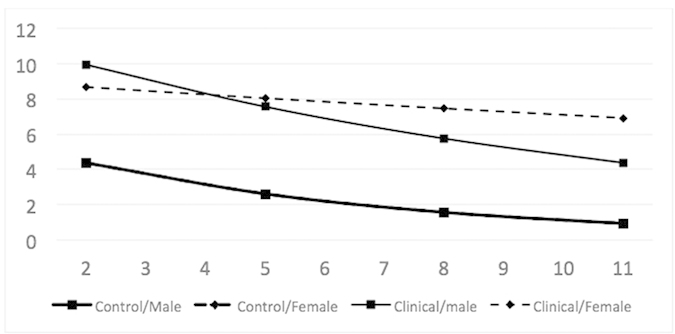
Modelled mean externalization growth curves by group and sex. Note: Curves represent mean developmental trajectories. Male and female trajectories in NWg overlay each other and appear as one trajectory.

As in the case of internalization, the model explained a substantial (although lesser) proportion of variance in externalization scores across waves and individuals; Model 1 captured 56% of variance, and Model 2 captured 58%. The difference between the model and the observed developmental trajectories was more pronounced here, especially in the case of Ug. Again, the effect of sex was significant in terms of information criteria, but the small sample prevented empirical determination of the nature of this effect. Based on theoretical expectations, we used only the interaction of sex with group, affecting intercept and rate of change and allowing females in Ug to differ in their development from the rest of the sample (Fig. 5).

### Relationship of EDI-3-RF scores with BMI and CBCL scales

The descriptive statistics by group and sex for EDI-3-RF total score are presented in Table 6. First we looked at differences in EDI-3-RF due to group and sex. A two-way ANOVA model explained 96% of variance in EDI-3-RF with all main effects (group: F(1, 156) = 4200; p < 0.001; gender: F(1, 156) = 123; p < 0.001), and the interaction was highly significant (F(1, 156) = 105; p < 0.001). In fact, the EDI-3-RF scores of NWg and Ug did not overlap at all, indicating that these scores are almost fully explained by group membership and sex. Although we attempted to utilize longitudinal information about BMI and CBCL scores, it added nothing to the prediction of EDI-3-RF scores.

## Discussion

The aim of this study was to examine the developmental trajectories of BMI and emotional-behavioral functioning of underweight children from age two years and to examine their risk of developing an eating disorder at early adolescence (age 11 years). Our first specific aim was to describe stability and change of BMI from age 2 to 11 in two groups of children (underweight and normal weight) of both sexes. Our results confirmed that children in the two groups showed different BMI trajectories over time. In particular, male and female subjects in NWg started from a higher BMI at two years of age and maintained this condition until T3 (age 8 years). Nevertheless, the trajectories of the two groups crossed around 11 years of age, with early adolescents who originally belonged to the underweight group catching up with their peers. Since few studies have focused on sex differences in BMI trajectories^41^, we addressed this issue and verified that boys in NWg have the highest BMI while males in Ug have the lowest BMI. In both NWg and Ug, girls’ scores are positioned between these extremes. Interestingly, sex differences disappeared in both groups at T4; this result is only partly consistent with Pryor *et al.*’s^42^ work, which reported no significant differences in the BMI patterns of males and females. In our data, the growth curve followed by NWg males differed from that of females, although they reached very similar BMI levels in early adolescence. In summary, both males and females in Ug moved from an underweight condition to a normal weight condition to reach an even higher BMI than early adolescents in NWg. This is a remarkable result, given that our study observed only subjects and families who did not pursue any treatment (either pharmacological or psychological) to alleviate children’s underweight (or to prevent or reduce symptoms or any other physical or psychological difficulty). Our results are also consistent with those of Boersma and Wit^43^ and other authors (44,45), showing that the vast majority of underweight children catch up with their peers at puberty or in early adolescence. Also worthy of note is the suggestion that the trend toward high BMI and obesity has been steadily increasing in developed countries in recent decades. For that reason, further studies will be needed to elucidate the mechanisms underpinning the shift from underweight to high BMI in underweight children. These mechanisms may include emotional-relational dimensions (e.g., quality of parent-child interaction)^19^, behavioral factors with a cognitive and/or biological basis (e.g., overeating)^46^, environmental factors (e.g., exposure to chemical compounds)^47^, and social and/or economic factors^48^,^49^.

Our second aim was to assess the emotional-behavioral functioning of male and female children in both groups over time. Previous research has focused mainly on externalizing symptoms in overweight children^50^. This can be explained by the fact that externalizing behavior is associated with many disturbances in both the child and its social environment, and that externalizing behavior is among the most prevalent mental health problems in childhood and adolescence^51^,^52^. Over the past 20 years, most studies on this topic have demonstrated that externalizing symptoms decline as children grow up (e.g., Bongers^53^ and Lahey^54^). While toddlers and preschool children often display a high level of externalizing behavior^51^, school-age children usually show a gradual decrease^55^,^56^. While internalizing problems in early childhood typically appear in the form of withdrawal, anxiety, fearfulness, and depression, externalizing problems usually take the form of explicit disruptive or oppositional behaviors such as aggression, defiance, and hyperactivity^51^,^57^,^58^,^59^; for these reasons, they seem to represent quite separate dysfunctional areas^60^,^61^,^62^. However, some studies have demonstrated that their trajectories may follow associated or interrelated directions, and the two problematic syndromes may present simultaneously in the same subject^63^. Our results show that while the underweight condition of children normalized over time, reaching the level of their peers, internalizing and externalizing problems in males and females in Ug remained higher than those of their peers at all points of assessment. In particular, while internalizing symptoms in boys and girls in Ug increased steadily over time, children in NWg showed low and slightly decreasing scores from T1 to T4. Largely consistent with the literature^55^,^64^,^65^,^66^, externalizing symptoms decreased in both groups over time, but Ug continued to show higher scores at all points of assessment. Taken together with the data on their physical condition, these findings on children’s trajectories in internalizing and externalizing problems suggest that psychological functioning in subjects with low BMI in early childhood do not indicate a more adaptive configuration over time. This conclusion should alert health professionals against focusing solely on the organic assessment of underweight children (which can normalize even in absence of any treatment) and instead to evaluate their emotional and behavioral functioning over the years, with due regard to both internalizing and externalizing problems, which, as we have verified, may remain problematic. According to Dodge and Pettit^67^, preventive interventions should start early in life before antisocial outcomes or other types of psychopathology become inevitable^68^. In support of this general idea, prior work has provided evidence that interventions during toddlerhood are successful in reducing externalizing^69^, internalizing^70^, and co-occurring behavioral and emotional problems^71^.

Our third aim was to assess the risk of eating disorder at 11 years in both of the study groups. Our research suggests that males and females in the Ug may be at increased risk of developing eating disorders in early adolescence or in later life. Indeed, Ug subjects scored higher than NWg subjects on total EDI-3-RF score. This result further supports the above recommendation to track the psychological functioning of underweight children over time, as our data suggest that these subjects are at a higher risk than their normal weight peers of developing an eating disorder.

This study has several strengths. First, it examines the developmental trajectories of BMI and emotional-behavioral functioning from early childhood to early adolescence, on which there is limited previous research^72^. Moreover, as well as observing the developmental trajectories of two groups (underweight and normal weight) in the manner of most previous studies on growth trajectories, we also described the differences between these. Second, rather than limiting our research to observing and describing these developmental trajectories, we examined the potential psychopathological risk to subjects, specifically assessing the risk of eating disorder. This clinical approach is rarely found in growth trajectory studies with community samples. Third, we employed widely used tools^57^ to measure the study variables, utilizing a selection of CBCL items on the basis of the previous literature (as described above), so ensuring that scores from T1 to T4 would be comparable for both internalizing and externalizing problems.

Nevertheless, the present research also has some limitations. First of all, we did not assess parents’ physical and psychological profiles, which precluded the possibility of correlating or associating parental characteristics with children’s profiles. For instance, some authors have suggested that parental weight, eating behavior, personality characteristics, and psychological profile may impact both children’s emotional-behavioral functioning and BMI^73^,^74^. This would limit the opportunity to identify parental factors that might be of relevance in the elaboration of intervention programs aimed to reduce underweight, maladaptive psychological functioning, and risk of eating disorder. Assessment of parents’ characteristics was included in the original protocol prepared in 2004, but the final research agreement with the schools and pediatricians who recruited the sample covered only measures of children. Additionally, we lacked a control sample for evaluation of the role of puberty in our subjects; we hypothesize that the observed catch-up among Ug subjects (which reached and surpassed NWg BMI scores) may be due to the sexual maturation of these youths (at least for females, all of whom reached menarche at T4). Taking account of other biological, individual, and sociocultural risk factors in conjunction with consideration of both risk and protective developmental processes could help to better explain our results^75^,^76^. For instance, callous–unemotional traits have been related to higher externalizing problems in children and to lower scores on internalizing problems^77^,^78^,^79^. The sample’s homogeneity in terms of race and geographical origin also precludes wide generalization of the results to a wider population.

We must also consider to what extent the change in BMI may have been due to the regression to the mean effect. In the case of BMI, there are two processes that cause regression to the mean. One is statistical, traditionally referred to as RTM effect; the other is intentional, in that caretakers deliberately try to adjust the child’s diet to achieve average (normal) BMI levels. Caretakers are fairly knowledgeable about what is normal, and their effort can be assumed to be proportional to the extremity of the child’s BMI. As this project is descriptive in nature, our data does not easily allow us to distinguish statistical RTM from intentional “striving for the mean”. Nevertheless, following Barnett *et al.*^80^, we attempted to estimate the probabilistic RTM effect by estimating the expected increase in mean BMI in a group selected at age 2 years, based on a single criterion: BMI < 2SD below mean. The range-restricted correlations in our sample are inadequate for the purpose of estimating within-person variance. Based on an Australian study^81^, the unrestricted BMIs show a correlation of 0.8 over a 3-year period; a conservative estimate for us, then, would be 0.4. Converted to the scale of BMI at age 11, the RTM effect would bring the scores up to almost 16, and so we would expect the Ug mean to be more than 1 SD below mean BMI. As Ug subjects in our sample grew to the population mean, we can assume that factors other than RTM are at play.

## Conclusions

These findings provide novel evidence of BMI and internalizing/externalizing developmental trajectories in underweight and normal weight children that have not previously been explored in such detail. Understandably, the literature to date has focused on the growth trajectories of children with high BMI and externalizing problems. However, the present results indicate a growing need for effective physical and psychological assessment of underweight children from the community in helping to design prevention programs to reduce the risk of onset of psychological difficulties such as eating disorders.

## Additional Information

**How to cite this article**: Cimino, S. *et al.* Developmental trajectories of body mass index and emotional-behavioral functioning of underweight children: A longitudinal study. *Sci. Rep.* **6**, 20211; doi: 10.1038/srep20211 (2016).

## Figures and Tables

**Table 1:** Descriptive statistics for study variables across the four waves.

Group	Wave	Age		BMI		CBCL INT		CBCL EXT	
		M	SD	M	SD	M	SD	M	SD
NWg	1	2.17	0.34	15.62	1.53	1.18	1.29	4.24	2.81
	2	5.09	0.52	16.06	1.00	1.60	1.59	3.29	2.95
	3	7.63	0.48	15.98	0.97	0.63	0.70	1.04	1.32
	4	11.23	0.20	18.57	1.20	0.86	0.91	1.05	1.40
Ug	1	2.75	0.70	13.56	1.35	4.14	2.73	8.36	3.44
	2	5.41	0.41	12.60	0.92	5.94	2.36	9.70	3.66
	3	7.64	0.47	13.40	0.70	7.05	2.90	5.20	2.65
	4	11.27	0.20	18.79	1.54	8.25	1.38	5.81	2.91

Note. N=80 in each cell.

**Table 2:** Correlations of BMI across waves (NWg above diagonal, Ug below).

	BMI 1	BMI 2	BMI 3	BMI 4
BMI 1		0.22	−0.11	−0.03
BMI 2	−0.07		−0.29	0.08
BMI 3	0.14	−0.09		0.13
BMI 4	−0.04	−0.03	0.12	

**Table 3:** Summary of BMI growth curve models (standard errors in parentheses).

Parameter	Model 1	*p*	Model 2	*p*
Intercept	16.371 (0.287)		18.129 (0.368)	
Age	−0.412 (0.099)	<0.01	−0.856 (0.128)	<0.01
Age^2	0.053 (0.007)	<0.01	0.079 (0.009)	<0.01
Ug	−0.005 (0.445)	0.99	−2.910 (0.588)	<0.01
Ug*Age	−1.123 (0.148)	<0.01	−0.327 (0.195)	0.09
Ug*Age^2	0.101 (0.011)	<0.01	0.053 (0.014)	<0.01
Female			−3.856 (0.544)	<0.01
Ug*Female			6.113 (0.843)	<0.01
Female*Age			1.001 (0.188)	<0.01
Female*Age^2			−0.059 (0.014)	<0.01
Ug*Female*Age			−1.698 (0.280)	<0.01
Ug*Female*Age^2			0.103 (0.020)	<0.01
Residual VAR	1.51 (0.08)	<0.01	1.35 (0.08)	<0.01
Information criteria				
−2LL	2073.0		2002.1	
AIC	2087.0		2028.1	
BIC	2118.2		2086.1	
Parameters	7		13	

Note. Group coded 1 for Ug and 0 for NWg; sex coded 1 for female and 0 for male. Variance of BMI across individuals and waves = 6.08.

**Table 4:** Correlations of internalization and externalization scores across waves. Normal weight group above diagonal, underweight below.

	INT 1	INT 2	INT 3	INT 4		EXT 1	EXT 2	EXT 3	EXT 4
INT 1		0.23	−0.01	−0.04	EXT 1		0.21	−0.08	−0.07
INT 2	0.34		0.15	0.05	EXT 2	0.71		0.03	−0.03
INT 3	−0.23	0.13		0.49	EXT 3	−0.18	0.02		0.80
INT 4	−0.30	0.11	0.61		EXT 4	−0.24	−0.04	0.84	

**Table 5:** Summary of internalization and externalization growth curve models.

Parameter	Internalization			Externalization				
	Model 1	*p*	Model 2	*p*	Model 1	*p*	Model 2	*p*
Intercept	0.404 (0.112)		0.553 (0.103)		1.821 (0.087)		1.822 (0.086)	
Age	−0.055 (0.016)	<0.01	−0.052 (0.015)	<0.01	−0.171 (0.014)	<0.01	−0.171 (0.014)	<0.01
Ug	0.911 (0.127)	<0.01	0.654 (0.120)	<0.01	0.508 (0.113)	<0.01	0.653 (0.132)	<0.01
Ug*Age	0.129 (0.018)	<0.01	0.124 (0.017)	<0.01	0.116 (0.017)	<0.01	0.081 (0.020)	<0.01
Female								
Ug*Female			0.242 (0.045)	<0.01			−0.263 (0.140)	0.06
Female*Age								
Ug*Female*Age							0.065 (0.019)	<0.01
Residual VAR	3.29	<0.01	3.37	<0.01	7.13	<0.01	6.88	<0.01
Information criteria								
−2LL	1343.4		1209.9		1397.4		1385.6	
AIC	1345.4		1211.9		1399.5		1387.6	
BIC	1349.9		1216.3		1403.9		1392.0	
Parameters	5		6		5		7	

Note. Group coded 1 for underweight group and 0 for normal weight group; sex coded 1 for female and 0 for male. Variance of internalization across individuals and waves = 11.8. Variance of externalization across individuals and waves = 16.2. Residual variance is the variance of residuals implied by the model.

**Table 6:** Descriptive statistics for EDI-3 RF by group and sex.

Group	Sex	M	SD	N
NWg	Male	0.71	0.20	40
	Female	0.75	0.22	40
	Total	0.73	0.21	80
Ug	Male	2.84	0.24	40
	Female	3.67	0.32	40
	Total	3.26	0.50	80
Total	Male	1.78	1.09	80
	Female	2.21	1.50	80
	Total	1.99	1.32	160
